# Far-field super-resolution ghost imaging with a deep neural network constraint

**DOI:** 10.1038/s41377-021-00680-w

**Published:** 2022-01-01

**Authors:** Fei Wang, Chenglong Wang, Mingliang Chen, Wenlin Gong, Yu Zhang, Shensheng Han, Guohai Situ

**Affiliations:** 1 Shanghai Institute of Optics and Fine Mechanics, Chinese Academy of Sciences, Shanghai, 201800 China; 2 Center of Materials Science and Optoelectronics Engineering, University of Chinese Academy of Sciences, Beijing, 100049 China; 3 Hangzhou Institute for Advanced Study, University of Chinese Academy of Sciences, Hangzhou, 310024 China; 4 CAS Center for Excellence in Ultra-intense Laser Science, Shanghai, 201800 China

**Keywords:** Imaging and sensing, Optical sensors

## Abstract

Ghost imaging (GI) facilitates image acquisition under low-light conditions by single-pixel measurements and thus has great potential in applications in various fields ranging from biomedical imaging to remote sensing. However, GI usually requires a large amount of single-pixel samplings in order to reconstruct a high-resolution image, imposing a practical limit for its applications. Here we propose a far-field super-resolution GI technique that incorporates the physical model for GI image formation into a deep neural network. The resulting hybrid neural network does not need to pre-train on any dataset, and allows the reconstruction of a far-field image with the resolution beyond the diffraction limit. Furthermore, the physical model imposes a constraint to the network output, making it effectively interpretable. We experimentally demonstrate the proposed GI technique by imaging a flying drone, and show that it outperforms some other widespread GI techniques in terms of both spatial resolution and sampling ratio. We believe that this study provides a new framework for GI, and paves a way for its practical applications.

## Introduction

Conventional imaging methods exploit the light reflected or scattered by an object to form its image on a two-dimensional sensor that has millions of pixels. However, ghost imaging (GI), an advanced imaging modality based on the second-order correlation of quantum or classical light, uses a single-pixel detector instead to record the reflected or scattered light, yielding a one-dimensional (1D) bucket signal^1–6^. In some cases, an additional position sensitive detector is required to measure the illumination patterns. Although neither detector directly records a resolvable image of the object, one can employ an intuitive linear algorithm to reconstruct its image by spatial correlating the acquired time-varying patterns and the synchronized bucket signal. As it uses a single-pixel detector to collect the photons that interact with the object, GI has significant advantages over conventional imaging modalities in terms of detection sensitivity, dark counts, spectral range, and cost efficiency^7,8^. In addition, with the aid of some prior information, e.g., sparsity, it is capable of sensing compressively during data acquisition^9,10^. Such enhancements can provide significance in low-light imaging where the photon counts are very low due to scattering or absorption losses as in medical imaging or remote sensing; and in non-visible waveband imaging where the availability of silicon-based sensor becomes expensive or impractical as in infrared or deep ultraviolet regime.

However, in GI, a large amount of single-pixel measurements is necessary because one sampling only contains a little information about the object. Specifically, to obtain an *N*-pixel image one needs at least *M* = *N* measurements to meet *β* = *M*/*N* = 100%, where *β* represents the sampling ratio (the Nyquist sampling criterion). In many applications such as remote sensing^10^, a rotating ground glass (RGG) is frequently used to generate speckle illumination patterns compared with other programmable modulation strategies, e.g., digital micromirror device^11^ owing to its high power endurance and cost efficiency. In this case one needs $M \gg N$ measurements to improve the signal-to-noise ratio (SNR) of the reconstructed image due to the overlap of different patterns^9^. This inevitably leads to a paradox between the number of pixels occupied by the object and the data acquisition time. In addition, the spatial resolution of GI is physically limited by the grain size of the speckle pattern on the object plane^12^. This is unfavorable for far-field imaging as the speckle grain becomes too large to distinguish the detailed structure of the object^13,14^. Thus, an intuitive and longstanding goal in the study of GI is to decrease *β* while retaining good resolution, so as to reduce the burden of data acquisition and produce better imaging visual effects. However, the consequential incomplete sampling strategy usually lead to ill-posedness in GI reconstruction. Thus, suitable prior assumptions are needed to compensate the missing information.

**Fig. 1:**
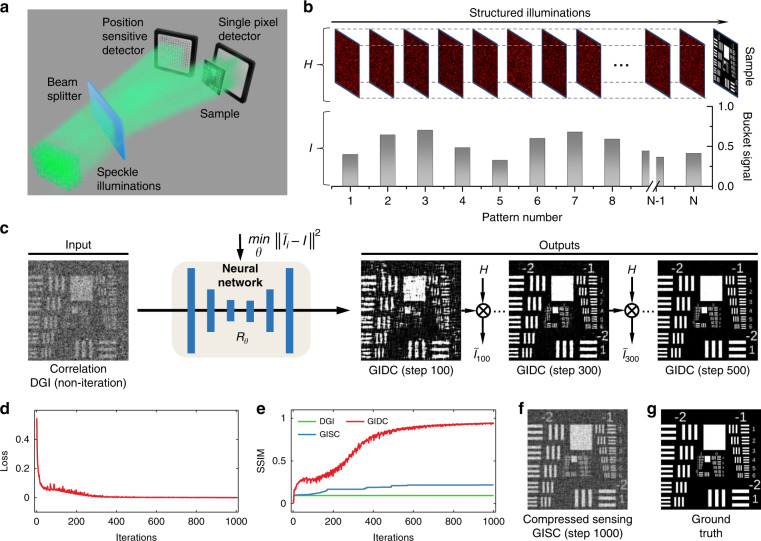
Overview of GIDC. **a** Sketch of the apparatus. Speckle illumination modes generated by the RGG were divided into a reference path that was directly measured by a pixelated camera and an object path that was measured by a single-pixel detector after interacting with the sample. **b** Raw speckle patterns *H* (top) and intensity sequence *I* (bottom) measured by the camera and the single-pixel detector, respectively. **c** Correlating *H* and *I* one can get a low-quality result by DGI especially when $\beta$ in our case is as low as 10%. Then, we feed it into the neural network ${{{\mathcal{R}}}}_\theta$ and keep it fixed. The output of the neural network is taken as the estimated object, which is then numerically multiplied with *H* to generate the estimated intensity sequence $\tilde I_i$ of *i*th step. We measure the mean square error (MSE) between *I* and $\tilde I_i$ as the loss function to guide the iteration of weights $\theta$ in ${{{\mathcal{R}}}}_\theta$. **d** MSE of *I* and $\tilde I_i$ along with the iteration steps *i* from 1 to 1000. **e** SSIM between the reconstruction results from DGI, GISC (**f**), GIDC, and ground truth (**g**) along with the iteration steps *i* from 1 to 1000

One popular approach is based on compressive sensing (CS). CS uses sparsity as a general prior assumption and has become a popular signal reconstruction framework^15–17^. It has been widely used in various imaging systems such as single-pixel cameras^11^ and compressive holography^18^. Specifically, given the measurements *y*, the CS technique usually reconstructs the object *x* by solving the following iteration problem:

1 $$\mathop {{{{{\mathrm{min}}}}}}\limits_x \parallel \Phi x - y\parallel _2^2 + \xi \parallel \Psi x\parallel _1$$

where $\Phi$ is the random measurement matrix and $\Psi$ is the transformation matrix that transforms *x* into a sparse domain such as discrete cosine transform (DCT) or wavelet. $\Psi x$ represents the corresponding transform coefficients regularized by the *l*_1_ norm with the regularization parameter $\xi$. Owing to the sparsity of the image of the object and the randomness of the illumination patterns, CS is also suitable for GI reconstruction. Such GI using sparsity constraint, or GISC for short, enables the reconstruction of high-quality and high-resolution image when *β* < 100%^7,9,13,19,20^ [Fig. 1f]. In the field of GI, CS has been used for resolution enhancement^21–23^, remote sensing^10^, 3D imaging^24^, and among many others^7,8,19^. However, it is still a challenging problem for GISC to operate well in the case when *β* is less than the Cramer–Rao bound^16,17,22^.

An alternative but increasingly important approach is deep learning that is based on data prior assumptions^25–27^. Specifically, it has shown that it allows robust GI reconstruction of high-quality images even when *β* < 10% with high computational efficiency^28–31^. Such GI based on deep learning (GIDL) technique uses a deep neural network (DNN) to learn from a large number of input–output data pairs so as to establish a mapping relationship among them. The experimental acquisition of such a huge training set is time consuming and laborious because one needs at least thousands of measurements for one data pair even for a 64 × 64 image in a proof-of-principle experiment. Though the neural network can be trained on simulation data^30^, the trained model only works well for the reconstruction of objects that resemble those in the training set. This challenge of generalization is one of the big issues that need to be addressed.

Recently, Ulyanov et al.^32^ proposed the deep image prior (DIP) framework that uses an untrained neural network as a constraint for image processing tasks such as denoising, inpainting, and super resolution. They demonstrated that a properly designed generator network architecture itself has an implicit bias towards natural images and thus can be used for solving ill-posed inverse problems^33^. The most significant advantage of DIP is that a generator network can be used without training beforehand, and thus eliminating the need for tens of thousands of labeled data. A similar concept has also been used for computational imaging, such as phase retrieval^34,35^, CS^36,37^, and diffraction tomography^38^.

Inspired by the idea of DIP, here we propose a new GI technique that incorporates the physical model of GI image formation into a DNN. We hypothesize that the image prior information introduced by an untrained DNN can be applied to achieve better GI reconstruction under much lower *β*. We term the proposed technique as GI using Deep neural network Constraint (GIDC). It utilizes an untrained DNN to generate high-quality and high-resolution results. The only input it requires are a 1D bucket signal sequence *I* from which one needs to reconstruct an image, together with the associated stack of illumination patterns *H*, which is easily accessible in a typical GI system [Fig. 1a]. The proposed GIDC technique is described as follows. First, we correlate the *H* [Fig. 1b, top] and *I* [Fig. 1b, bottom] by differential ghost imaging (DGI)^39,40^ and obtain a rough reconstruction of the image. Second, we feed the resulting DGI reconstruction into a randomly initialized neural network (untrained). Third, we take the output of the neural network as an estimation of a high-quality GI image and use it to calculate a bucket signal *I* by using a GI image formation model. Finally, we update the weights of the neural network to minimize the error between the measured and estimated bucket signal [Fig. 1c]. Along with the error reduction [Fig. 1d], the output of the neural network also converges to a good-quality image [Fig. 1e]. Compared with conventional DGI and GISC [Fig. 1f], the proposed strategy dramatically increases the quality and resolution of GI under much lower sampling ratio *β*. Compared with those state-of-the-art deep-learning-based methods, GIDC does not need to train on any labeled data and thus is more flexible and does not bias towards a specific distribution. Specifically, our contributions include:

- We demonstrate that GIDC can reconstruct a dramatically high SNR GI image at a very low sampling ratio *β*.
- We demonstrate that GIDC can enhance the resolution of the reconstructed image even when the speckle grain size is larger, suggesting its potential to break the diffraction limit.
- We perform a comparative study on the base of a number of challenging real-world scenarios including a flying drone and synthesized dataset, and demonstrate that GIDC outperforms other widespread GI methods, including DGI, GISC, and GIDL.

## Results

### Sampling ratio

**Fig. 2:**
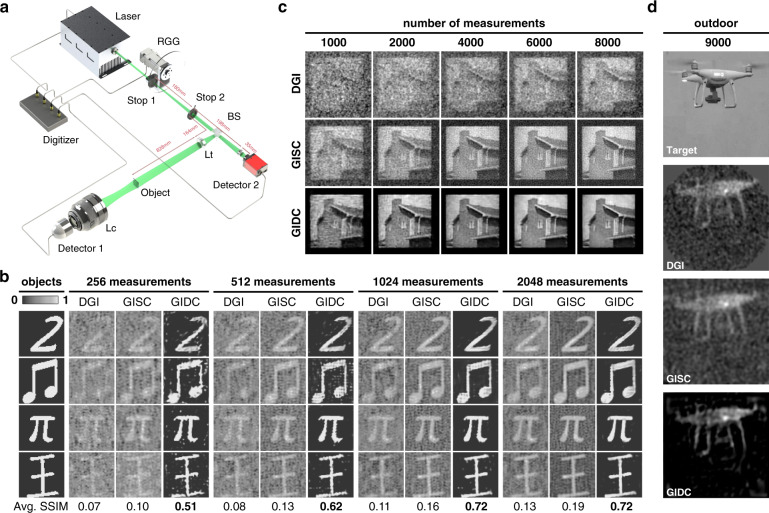
Experimental comparisons of DGI, GIDC and the proposed GIDC in terms of both sampling ratio and reconstruction SNR. **a** Schematic diagram of the experimental setup. **b** Experiment results for binary objects. Each row in the group represents the results of the same object reconstructed by different methods, while each column represents the results of different objects reconstructed by the same method. The GIDC iteration step is $S = 500$. The pixel resolution is $N = 64 \times 64$. **c** Experiment results for a grayscale object. Each row shows the results obtained by different methods with different measurement time changes by column. $S = 1000$, $N = 128 \times 128$. **d** Experiment results on a flying drone. $S = 200$, $N = 128 \times 128$

We built a typical Pseudothermal GI system [Fig. 2a] for data acquisition. Here we show the reconstruction results of different objects using different methods at different sampling ratios. The first group of results is plotted in Fig. 2b. One can clearly see that all the binary objects have been successfully reconstructed by GIDC, with the number of measurements as low as 256 (*β* = 6.25%). We also take DGI^39,40^ and GISC^13^ for comparison. For all the cases (different objects and *β* settings), GIDC outperform DGI and GISC both in terms of visual appearance and quantitative evaluation index (SSIM). We observe the same results in the cases that the object is in grayscale [Fig. 2c]. One can clearly see that the clean and high-contrast images reconstructed by GIDC, whereas the ones recovered by DGI and GISC are dirty or even corrupted by strong noise in particular when *β* is low (see first two columns in Fig. 2c).

We also conducted an outdoor experiment to demonstrate the effectiveness of GIDC. The data were acquired by using a homemade GI LiDAR system^41^. The imaging target [Fig. 2d, top] is a flying drone (DJI, Phantom 4) hovering in the air, 50 m away from the GI LiDAR system. The main results are plotted in Fig. 2d. One can clearly see that the GIDC can successfully reconstruct the shape of the drone with very high contrast. The size of the reconstructed image is 128 × 128, meaning that the sampling ratio *β* = 9000/16,384 ≈ 55%. The reconstructed image by DGI and GISC plotted as well for comparison. One can see that the image reconstructed by DGI or GISC is corrupted by noise, and the contrast is low.

### Resolution

**Fig. 3:**
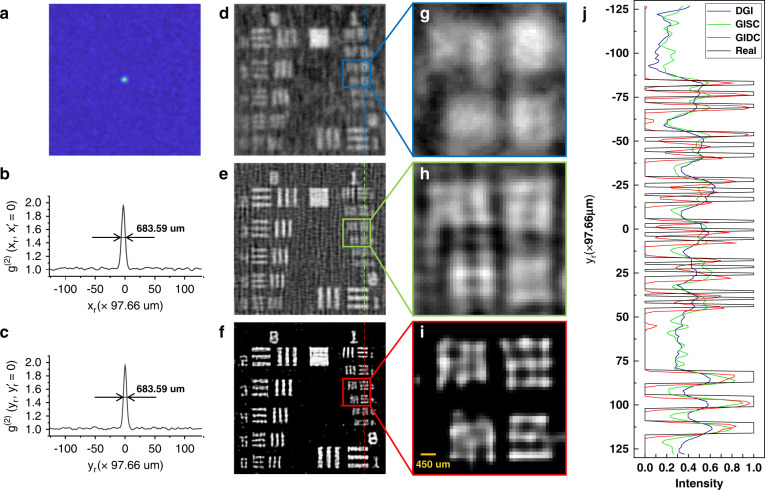
Experiment results for USAF resolution target. From the normalized correlation function of intensity distribution on the reference detection plane (**a**), one can get the cross-section curves at ($x_r,y_r = 128$) (**b**), and ($x_r = 128,y_r$) (**c**) so as to measure the corresponding FWHM and demonstrate the diffraction limit of the GI system. **d**–**f** Results obtained by DGI, GISC, and GIDC, respectively. **g**–**i** Zoomed-in images of different ROIs of (**d**–**f**). **j** Cross-sectional profile of dash line shown in (**d**–**f**). The iteration steps used for GIDC is 500; 9216 measurements were used for the reconstruction. The pixel resolution is $256 \times 256$ pixels and the spatial resolution is 7 binned pixels

We also experimentally demonstrated the spatial resolution that GIDC can offer. It is known that as an imaging method based on the second-order (intensity) correlation of light, the spatial resolution of GI is theoretically limited by the width of the mutual correlation function of the illumination speckle patterns, measured at the object plane^42^. According to this, we first calculated the normalized correlation function [Fig. 3a] of the recorded speckle patterns^43^, namely

2 $$g^{(2)}\left( {x_r,x_r^\prime = 0} \right) = \frac{{\left\langle {H_1(x_r)H_2(x_r^\prime = 0)} \right\rangle }}{{\left\langle {H_1(x_r)} \right\rangle \left\langle {H_2(x_r^\prime = 0)} \right\rangle }}$$

Then, we measured the full-width at half-max (FWHM) to estimate the value of the speckle grain size on the object plane. We found that it occupies 7 binned pixels in both the horizontal and vertical directions [Fig. 3b, c], suggesting that the diffraction limit of our experimental GI system is 683.59 μm. More details about the system configuration toward the GI system can be found in the section “Methods and Materials.”

A USAF resolution target was used to test the resolution of different GI reconstruction methods. The main results are plotted in Fig. 3d–i. As expected, the image reconstructed by DGI suggests that the elements in Group 0 Element 5 are not resolvable because the linewidth (629.96 μm) is smaller than the diffraction limit (683.59 μm). It becomes a little bit better by using GISC, where some elements with their linewidth smaller than the diffraction limit (Group 1 Element 1, 500 μm) can be distinguished. Evidentially, the proposed GIDC has the best performance in terms of both linewidth and sharpness exhibited in the reconstructed image. As shown in Fig. 3g–i, the line pairs in Group 1 Element 4 with the linewidth of 353.55 μm can be successfully reconstructed by GIDC, but neither DGI nor GISC achieves the same performance. This suggests that the proposed GIDC has the capability of enhancing the resolution by a factor of about 2 (683.59/353.55 = 1.93) with respect to the diffraction limit. More evidence can be found in Fig. 3j. In addition to the advantages of resolution, the image reconstructed by GIDC has much higher contrast as evidenced by the clean background.

## Discussion

In this section, we make some more in-depth discussions on the performance of GIDC in comparison to DGI and GISC. GIDL trained on two different datasets were also considered. For the sake of quantitative evaluation, we examine on simulation data in this section.

### Accuracy

**Fig. 4:**
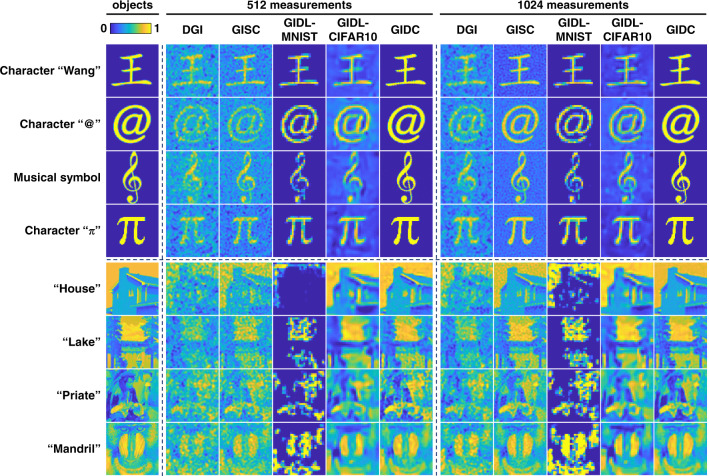
Comparison of different GI reconstruction methods under different $\beta = 512/4096 = 12.5\%$ and $\beta = 1024/4096 = 25\%$. For GIDL, 50,000 data pairs generated by MNIST and Cifar10 associated with different *β*s were used to train the neural network, respectively. The network architecture is the same as that we used in GIDC. The models were trained 60 epochs with a learning rate of 0.001. In this way, four trained models associated with different training sets and *βs* were obtained. The iteration step of GIDC is 1000. The image size is 64 × 64

Two different *β* settings were studied here, i.e., *β* = 12.5% and *β* = 25%, corresponding to the number of measurements *M* = 512 and 1024, respectively. The results are shown in Fig. 4. Apparently, the images reconstructed by GIDC have the best fidelity for all the sample objects we studied here. In the case of $\beta = 12.5\%$, we observed that the reconstructed grayscale images are not as good as the reconstructed binary images even using GIDC. This is probably because a grayscale image contains too much unknown information to be determined, and it seems to be unfeasible to achieve a good reconstruction with a small sampling ratio. However, the reconstructed images are much better when $\beta = 25\%$, which is in consistence with the optical experimental results shown in Fig. 2. In order to quantitatively evaluate the results obtained by different methods, we calculated the SSIM value for each reconstructed image with respect to the corresponding ground truth. The SSIMs are listed in Table 1. It is clearly seen that GIDC has the highest metrics values in most of the cases, suggesting that the reconstruction accuracy of GIDC outperforms the others. The performance of GIDL, however, depends strongly on the training set and the task in hand. For instance, relatively good performance can be achieved when using GIDL trained on MNIST to reconstruct binary characters. In contrast, the reconstructed images are severely corrupted in the cases of grayscale due to the limited generalization. Although this can be slightly relieved by training it on an alternative dataset such as Cifar10^44^, it affects the accuracy of the reconstructed binary characters images as suggested by results shown in the fifth and tenth columns in Fig. 4. By contrast, GIDC is a general method that can be used to reconstruct different types of objects usually with a high accuracy and a low *β*.

**Table 1:** The metrics of different GI reconstruction methods on SSIM when $\beta = 512/4096 = 12.5\%$ and $\beta = 1024/4096 = 25\%$

Number of measurements	512					1024				
Objects\Methods	DGI	GISC	GIDL-MNIST	GIDL-CIFAR10	GIDC	DGI	GISC	GIDL-MNIST	GIDL-CIFAR10	GIDC
Character “Wang”	0.158	0.298	0.819	0.303	**0.870**	0.209	0.423	0.809	0.342	**0.917**
Character “@”	0.109	0.187	0.858	0.220	**0.971**	0.130	0.252	0.866	0.226	**0.978**
Musical symbol	0.109	0.179	0.827	0.177	**0.898**	0.131	0.232	0.843	0.215	**0.889**
Character “$\pi$”	0.089	0.138	0.836	0.193	**0.993**	0.100	0.208	0.866	0.237	**0.995**
“House”	0.179	0.397	0.016	0.452	**0.653**	0.218	0.502	0.102	0.470	**0.790**
“Lake”	0.177	0.431	0.075	0.423	**0.606**	0.254	0.634	0.119	0.468	**0.697**
“Priate”	0.267	**0.513**	0.130	0.412	0.504	0.337	0.657	0.189	0.493	**0.726**
“Mandril”	0.229	0.463	0.132	0.407	**0.641**	0.332	0.573	0.196	0.476	**0.712**

Bold values indicates the highest quantitative metrics (SSIM)

### Resolution

**Fig. 5:**
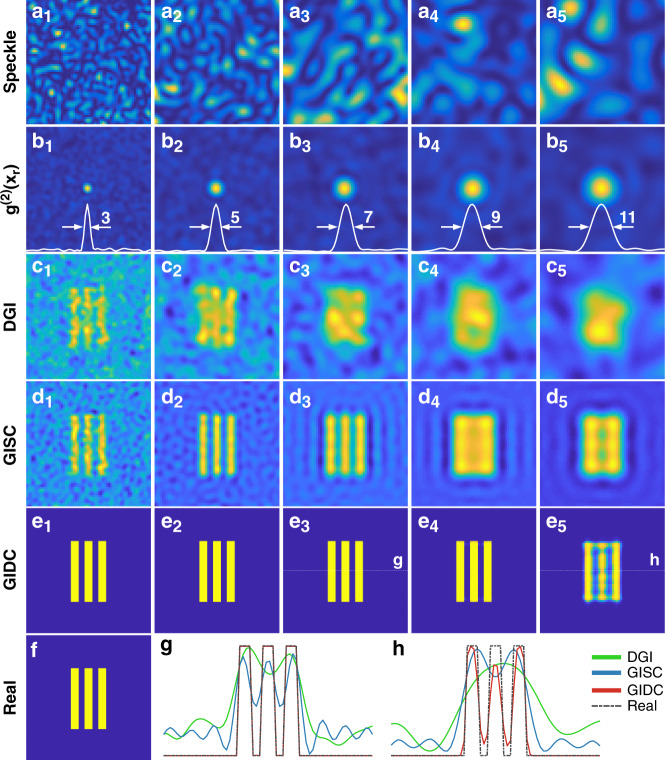
Comparison of GI resolution using different reconstruction algorithms. **a**_**1**_–**a**_**5**_ One of the speckle patterns used for generating the bucket signal. **b**_**1**_**–b**_**5**_ The mutual correlation function was used for estimating the speckle grain size. From left to right the FWHM gradually increased. The unit is μm. **c**_**1**_**–c**_**5**_, **d**_**1**_**–d**_**5**_, and **e**_**1**_**–e**_**5**_ The DGI, GISC, and GIDC results using the corresponding speckles. **f** The real object. **g**, **h** Line plots show image intensities along the dashed lines in **e**_**3**_ and **e**_**5**_, respectively

Here we will analyze the experiment result on resolution enhancement shown in the section “Resolution.” First we compare the resolution of the images reconstructed by GIDC and other widespread GI algorithms from the same set of simulation data. We generated five groups of illumination speckles [Fig. 5a1–a5] with the grain size $x_s = \lambda z/D$ varying from 3 to 11 μm [Fig. 5b1–b5] to encode the object which was a triple-slit pattern shown in Fig. 5f. We set $\beta = 410/4096 \approx 10\%$. We found that the DGI cannot distinguish the slits well when $x_s \,>\, 5$ μm. As expected, GISC can enhance the resolution. As evidenced in Fig. 5d3, the slit pattern can still be recognized when $x_s$ is as large as 7 μm. However, GISC fails when $x_s \ge 9$ μm. By contrast, the proposed GIDC reconstructs an almost perfect image under the same condition. Even when $x_s$ is as large as 11 μm, the GIDC still provide a very good result [Fig. 5e5]. The cross-section of the reconstructed image when $x_s = 7$ μm and $x_s = 11$ μm was plotted in Fig. 5g, h, respectively. From the results, one can clearly conclude that the proposed GIDC can provide dramatically resolution enhancement compared with DGI and GISC, in high consistence with the experimental results presented in Fig. 3.

Note that in the studies of phase imaging using untrained neural networks^34,35^, we did not observe such a phenomenon of resolution enhancement. So it must have something to do with the imaging modality of GI. There are three unique features that GI possesses in comparison to phase imaging. First, the object is illuminated by a random beam. Second, the light scattered from the object is recorded with a bucket detector. Third, GI relies on the second-order correlation of the light field^45^, whereas phase imaging relies on the first order. Each implementation of the random illumination can shift some of the high-spatial frequency components to the lower band^46^. This means that the associated information beyond the diffraction limit can be efficiently encoded and transmitted to the detector. A similar concept has been introduced in microscopy to achieve super resolution as well^47^. In the case of GI, however, the decoding of those high-frequency components is not so trivial due to the fact that they are highly compressed in the 1D bucket signal. Indeed, as shown in Fig. 5, none of those widespread GI algorithms can do this job. In contrast, GIDC endeavors to find a feasible solution that can reproduce the acquired bucket signal. Such a feasible solution has to contain those high-frequency components encoded in the bucket signal in order to decrease the loss function.

### Robustness

The robustness is evaluated by examining the effect of noise in the detection to the reconstructed image. There are different kinds of noise in the detection process^48^, but the noise effect can be modeled as an additive Gaussian distribution with the standard deviation $\delta$ as a whole^20,49^. Thus, one can define the detection SNR (dSNR) as follows^50^:

3 $${{{\mathrm{dSNR}}}} = 10{{{\mathrm{log}}}}_{10}\frac{{\left\langle I \right\rangle }}{\delta }$$

to describe the degradation of the detected signal. Two cases were examined in our studies. In the first case, we fixed the dSNR to 26 dB, and see how the reconstructed image would be under different sampling ratio conditions. In the second one, we fixed the sampling ratio to be 60% for different dSNRs. In this analysis, eight standard grayscale images (Supplementary Fig. S1) were used as the target. We again used SSIM to measure the quality of the reconstructed image from the contaminated bucket signal. The results are plotted in Supplementary Fig. S2; one can clearly see that GIDC has the best performance among all the three, in particular when the noise level is high. For DGI, the SSIM value of the reconstructed image is linearly increased with the sampling ratio *β* as the SNR of the reconstructed image is linearly proportional to the number of measurements^39,40^. In addition, we observed that the averaged SSIM in this case is around 0.46 when $\beta = 60\%$. This noise-independence effect is highly consistence with the theoretical prediction^39,40^. On the contrary, GISC is more sensitive to the detection noise^15^ as the SSIM drops from 0.862 to 0.544 when the dSNR is decreased from 30 to 22 dB. Some visualization results can be found in Supplementary Fig. S3.

### Priors

The effect of priors is also examined here. Two types of priors were used in GIDC, the physical prior, i.e., DGI, and the total variation (TV) regularization. Here we analyze the effect of DGI and TV independently and in combination. When either DGI or TV is not used, the associated SSIM values are plotted as the bars in green and turquoise, respectively, in Supplementary Fig. S2. One can see that, in all the cases, the SSIM values are slightly less than the one associated with GIDC (orange). This suggests that the use of priors does have contribution to the quality of the reconstructed image. This can be more clearly seen by the yellow bars, which are associated with the cases that neither of them was used. But even in this case the reconstructed image is still far better than the one obtained from DGI alone, suggesting that the GIDC framework has good robustness performance. Some visualization results can be found in Supplementary Fig. S3.

### Computational efficiency

It is necessary to compare the computational time for different approaches. Different image sizes were considered when *β* is set to 6.25%. Compared with DGI and GISC, GIDC provides the best results in terms of both visualizations [Supplementary Fig. S4a] and quantitative metrics [Supplementary Fig. S4c] under all pixel-resolution settings. However, as shown in Supplementary Fig. S4b, GIDC needs the longest time to optimize. For a 128 × 128 image, it needs about 5 min to restore a feasible result, while DGI and GISC only needs 0.221 and 12.29 s, respectively. Thus, the previous GISC and our GIDC are both not suitable for real-time applications, at least on the current computing platform. Despite this, for applications that allow post-processing offline but require fast data acquisition, GIDC yields the highest image fidelity at the lowest sampling ratio. The reconstructed image is associated with a SSIM value of 0.9 even when *β* is down to 6.25%. We also noticed that the computational time dramatically increases along with the increase of the image size. There are mainly two reasons for this. First, the width of the network will increase accordingly to accept the image as its input, process it and produce an output. Thus, it takes more time to forward infer during each iteration. Second, the size of the measurement matrix *H* that is used to generate the estimated bucket signal will increase. Thus, it takes more time to calculate the gradient and update the network parameters.

There are several strategies that one can take into account to improve the GIDC computational efficiency. These include better design of the neural network architecture, the implementation of depth-wise convolution^51^, the employment of better initialization^52^ and learning^53^ strategy. In addition, from a practical application point of view, the implementation of GIDC on a faster computing platform together with hardware speedup by using multiple GPUs will also help to significantly increase the computational efficiency.

## Methods and materials

### Formation of the reconstruction algorithm

For an object $O(x_t,y_t)$, the measurements of the pseudothermal GI system are the 1D bucket signal

4 $$I_m \propto \mathop {\sum}\limits_{x_t,y_t} {H_m(x_t,y_t)O(x_t,y_t)}$$

measured by a single-pixel detector in the test arm, and the corresponding stack of random illumination patterns $H_m(x_t,y_t)$, where $m = 1,2, \ldots ,M$, measured by a high-resolution camera in the reference arm. The conventional GI algorithm reconstructs the object image by computing the intensity correlation between *H*_*m*_ and *I*_*m*_

5 $$O_{GI} = \left\langle {H_mI_m} \right\rangle - \left\langle {H_m} \right\rangle \left\langle {I_m} \right\rangle$$

where $\left\langle \cdot \right\rangle$ denotes the ensemble average approximately defined as $H_m = \frac{1}{M}\mathop {\sum}\nolimits_{m = 1}^M {H_m}$ and $I_m = \frac{1}{M}\mathop {\sum}\nolimits_{m = 1}^M {I_m}$.

For DGI^39,40^, one uses $S_m = \mathop {\sum }\limits_{x_t,y_t} H_m(x_t,y_t)$ to normalize the illumination patterns so as to improve the SNR

6 $$O_{DGI} = \left\langle {H_mI_m} \right\rangle - \frac{{\left\langle {H_m} \right\rangle }}{{\left\langle {S_m} \right\rangle }}\left\langle {S_mI_m} \right\rangle$$

For the proposed GIDC, the reconstruction of the object image is formulated as the following objective function

7 $${{{\mathcal{R}}}}_{\theta ^ \ast } = {{{\mathrm{argmin}}}}_{\theta \in \Theta }\mathop {\sum}\limits_{m = 1}^M \parallel {\sum} {H_m{{{\mathcal{R}}}}_\theta (O_{DGI}) - I_m} \parallel ^2$$

where ${{{\mathcal{R}}}}_\theta$ is the DNN defined by a set of weights and biases parameters $\Theta$. The goal of GIDC is to find a good configuration $\theta ^ \ast \in \Theta$ for the neural network that forces its output $O_{GIDC} = {{{\mathcal{R}}}}_{\theta ^ \ast }(O_{DGI})$ to produce a 1D sequence $\tilde I$ according to the GI image formation physics (Eq.4) that resembles the experimentally acquired bucket signal *I*. As it is an ill-posed problem, especially when $M \ll N$, there are in principle an infinite number of configurations that satisfies the objective function. Therefore, it is necessary to add prior information about the object so as to select a feasible solution from all the configurations. For example, in GISC, the prior information is about an assumption that the object is sparse in a certain domain. Different from GISC, the proposed GIDC is based on an untrained DNN prior. Although the theory for this has yet to be perfected, existing works has empirically suggested that a properly designed DNN with randomly initialized weights has an inherent bias toward natural images^32,34–38^. We thus hypothesize that the DNN prior can be used to solve the ill-posed problem described by Eq. (7). We also argue that adding a conventional regularization terms such as the TV^38^ in the GIDC framework would help improving the reconstruction results. So the final objective function (loss function) of GIDC is reformulated as follows:

8 $${{{\mathcal{R}}}}_{\theta ^ \ast } = {{{\mathrm{argmin}}}}_\theta \parallel H{{{\mathcal{R}}}}_\theta (O_{DGI}) - I\parallel ^2 + \xi {{{\mathcal{T}}}}[{{{\mathcal{R}}}}_\theta (O_{DGI})]$$

where ${{{\mathcal{T}}}}$ stands for TV and $\xi$ is its strength.

For comparison, it is worthy of pointing out that GIDL uses a DNN as well. But it attempts to learn the mapping function ${{{\mathcal{R}}}}_\theta$ from a large number of labeled data pairs in the training set $S_T = \{ (O_{DGI}^k,O^k)|k = 1,2, \ldots ,K\}$, by solving

9 $${{{\mathcal{R}}}}_{\theta ^ \ast } = {{{\mathrm{argmin}}}}_{\theta \in \Theta }\parallel {{{\mathcal{R}}}}_\theta (O_{DGI}^k) - O^k\parallel ^2,\forall (O_{DGI}^k,O^k) \in S_T$$

GIDL learns to map the low-quality reconstructed images to a high-quality ones from the statistics of the training set $S_T$. Once trained, the neural net can be used directly to reconstruct objects that are similar with those in $S_T$.

By contrast, GIDC learns the mapping function through updating the weights and biases $\theta$ in the neural network to minimize the model-based fidelity term, which can be seen as an interplay between the GI physical model *H* and the DNN ${{{\mathcal{R}}}}_\theta$. In this way, one can obtain a feasible solution $O_{GIDC} = {{{\mathcal{R}}}}_{\theta ^ \ast }(O_{DGI})$ without using any training data. That is to say, GIDC is an untrained method and does not bias toward any particular dataset. We note that the input of the neural network used in GIDC can be a coarse image recovered by any conventional GI algorithms^20,34,39,40,46^ or even random noise^32,35,38^, here we use the result of DGI for convenience.

### Network architecture and hyper parameters

The network architecture we employed in this work was derived from the U-net^54^. More details of the network structure are provided in Supplementary Fig. S5. We adopted the Adam optimizer with a learning rate of ${\upalpha} = 0.05$, $\beta _1 = 0.5$, $\beta _2 = 0.9$, and ${\it{\epsilon }} = 10^{ - 9}$ to update the weights in the neural network. We also used an exponential decay with a decay rate of 0.9 and decay steps of 100. The momentum and epsilon parameters in the batch normalization were 0.99 and 0.001, respectively. The leak parameter of Leaky ReLU was 0.2. The regularization parameter of the TV was 10^−10^. The code was run on a computer with an Intel Xeon CPU E5-2696 V3, 64 GB RAM, and an NVIDIA Quadro P6000 GPU. The main progress is illustrated in Algorithm 1. For the sake of comparison, we use the same network model for GIDC and GIDL. We also released our code at https://github.com/FeiWang0824/GIDC.

**Figure:**
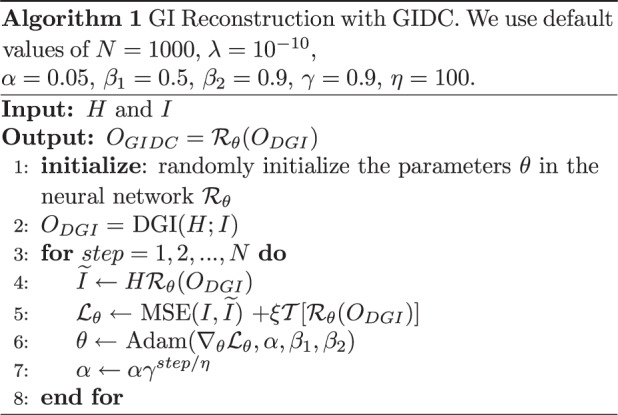

### Experimental details

Figure 2a presents the optical system we built for the experimental demonstration. $L_t$, $L_r$ and $L_c$ are lenses with focal length of 136.8, 30, and 75 mm, respectively. Detector 1 works as a single-pixel detector, whereas Detector 2 is a high-resolution camera. The light source is a solid-state pulsed laser with a $\lambda = 532$ nm center-wavelength, and a 10 ns pulse width at a repetition rate of 1 kHz. The pulsed beam emitted from the laser irradiated a RGG to produce pseudothermal light. The beam diameter on the RGG was $D$ and can be adjusted by an optical stop (stop1). The distance between the RGG and the other optical stop (stop2) is about $z = 180$ mm. The shape of stop2 is a square with a side length equal to 5 mm. Owing to the RGG, a speckle field is fully developed at the plane of stop2. Then the speckle field is divided by a beam splitter into a test and a reference arms. In the test arm, we use an imaging lens $L_t$ to project the speckle field at the stop2 plane to the surface of the object. The side length of the objects is about $L = 25$ mm (the magnification of $L_t$ is 5). The transmitted light is collected by a lens $L_c$ (Nikon AF-S NiKKOR 85 mm f/1.4 G) and finally recorded by a single-pixel detector (in our experiment, we actually used an AVT F504B camera to record the transmitted intensity, and generated the bucket signal by summing all the pixel values). In the reference arm, we use an image detector 2 (AVT F504B, with the pixel size $p_s$ of 3.45 μm) mounted with an imaging lens $L_r$ to take a high-resolution image of the speckle pattern on the stop2 plane.

Three different types of objects were used to test the GIDC performance, i.e., transparent slices of various characters with binary value and of natural scenes in grayscale (a film after exposure a standard test image “house”), and a physical USAF resolution chart. The spatial resolution of our GI system can be adjusted by changing $D$ as $z$ is fixed through $x_s = \lambda z/D$. In addition, different pixel resolution *N* can be obtained by setting different resize factor $q = \sqrt {L/(\sqrt N p_s)}$ so that the binned pixel size is $q^2p_s$. We set the resize factor of 10.64, 7.52, and 5.32 for binary characters, grayscale object, and USAF resolution chart to meet the pixel resolution of 64, 128, and 256, respectively. Then the binned pixel size is 390.63, 195.31, and 97.66 μm, respectively. For the experiment of USAF resolution chart, we set *D* = 0.70 mm to meet the spatial resolution of 683.59 μm (683.59/97.66 = 7 binned pixels).

## Supplementary information

Supplementary Material

## References

[1] Pittman TB (1995). Optical imaging by means of two-photon quantum entanglement. Phys. Rev. A.

[2] Strekalov DV (1995). Observation of two-photon “ghost” interference and diffraction. Phys. Rev. Lett..

[3] Gatti A (2004). Ghost imaging with thermal light: comparing entanglement and classical correlation. Phys. Rev. Lett..

[4] Cheng J, Han SS (2004). Incoherent coincidence imaging and its applicability in X-ray diffraction. Phys. Rev. Lett..

[5] Erkmen BI, Shapiro JH (2010). Ghost imaging: from quantum to classical to computational. Adv. Opt. Photonics.

[6] Moreau PA (2018). Ghost imaging using optical correlations. Laser Photonics Rev..

[7] Edgar MP, Gibson GM, Padgett MJ (2019). Principles and prospects for single-pixel imaging. Nat. Photonics.

[8] Gibson GM, Johnson SD, Padgett MJ (2020). Single-pixel imaging 12 years on: a review. Opt. Express.

[9] Katz O, Bromberg Y, Silberberg Y (2009). Compressive ghost imaging. Appl. Phys. Lett..

[10] Zhao CQ (2012). Ghost imaging lidar via sparsity constraints. Appl. Phys. Lett..

[11] Duarte MF (2008). Single-pixel imaging via compressive sampling. IEEE Signal Process. Mag..

[12] Ferri F (2005). High-resolution ghost image and ghost diffraction experiments with thermal light. Phys. Rev. Lett..

[13] Gong WL, Han SS (2015). High-resolution far-field ghost imaging via sparsity constraint. Sci. Rep..

[14] Li ZP (2020). Super-resolution single-photon imaging at 8.2 kilometers. Opt. Express.

[15] Candés EJ, Romberg JK, Tao T (2006). Stable signal recovery from incomplete and inaccurate measurements. Commun. Pure Appl. Math..

[16] Donoho DL (2006). Compressed sensing. IEEE Trans. Inf. Theory.

[17] Eldar, Y. C. & Kutyniok, G. *Compressed Sensing: Theory and Applications* (New York: Cambridge University Press, 2012).

[18] Brady DJ (2009). Compressive holography. Opt. Express.

[19] Han SS (2018). A review of ghost imaging via sparsity constraints. Appl. Sci..

[20] Bian LH (2018). Experimental comparison of single-pixel imaging algorithms. J. Optical Soc. Am. A.

[21] Gong WL, Han SS (2012). Experimental investigation of the quality of lensless super-resolution ghost imaging via sparsity constraints. Phys. Lett. A.

[22] Li WW (2019). Single-frame wide-field nanoscopy based on ghost imaging via sparsity constraints. Optica.

[23] Amitonova LV, de Boer JF (2020). Endo-microscopy beyond the Abbe and Nyquist limits. Light.: Sci. Appl..

[24] Sun MJ (2016). Single-pixel three-dimensional imaging with time-based depth resolution. Nat. Commun..

[25] Goodfellow, I., Bengio, Y. & Courville, A. *Deep Learning* (Cambridge: MIT Press, 2016).

[26] LeCun Y, Bengio Y, Hinton G (2015). Deep learning. Nature.

[27] Barbastathis G, Ozcan A, Situ G (2019). On the use of deep learning for computational imaging. Optica.

[28] Lyu M (2017). Deep-learning-based ghost imaging. Sci. Rep..

[29] He YC (2018). Ghost imaging based on deep learning. Sci. Rep..

[30] Wang F (2019). Learning from simulation: an end-to-end deep-learning approach for computational ghost imaging. Opt. Express.

[31] Higham CF (2018). Deep learning for real-time single-pixel video. Sci. Rep..

[32] Lempitsky, V., Vedaldi, A. & Ulyanov, D. Deep image prior. *Proceedings of 2018 IEEE/CVF Conference on Computer Vision and Pattern Recognition (CVPR)* (Salt Lake City, UT, USA: IEEE, 2018).

[33] Dittmer S (2020). Regularization by architecture: a deep prior approach for inverse problems. J. Math. Imaging Vis..

[34] Wang F (2020). Phase imaging with an untrained neural network. Light.: Sci. Appl..

[35] Bostan E (2020). Deep phase decoder: self-calibrating phase microscopy with an untrained deep neural network. Optica.

[36] Van Veen, D. et al. Compressed sensing with deep image prior and learned regularization. Preprint at arXiv: 1806.06438 (2018).

[37] Heckel, R. & Soltanolkotabi, M. Compressive sensing with un-trained neural networks: gradient descent finds the smoothest approximation. *Proceedings of the 37th International Conference on Machine Learning* (eds III, Hal, D. and Singh, A.). **119**, 4149–4158 http://proceedings.mlr.press/v119/heckel20a/heckel20a.pdf (PMLR, 2020).

[38] Zhou KC, Horstmeyer R (2020). Diffraction tomography with a deep image prior. Opt. Express.

[39] Gong WL, Han SS (2010). A method to improve the visibility of ghost images obtained by thermal light. Phys. Lett. A.

[40] Ferri F (2010). Differential ghost imaging. Phys. Rev. Lett..

[41] Wang CL (2018). Airborne near infrared three-dimensional ghost imaging LiDAR via sparsity constraint. Remote Sens..

[42] Bromberg Y, Katz O, Silberberg Y (2009). Ghost imaging with a single detector. Phys. Rev. A.

[43] Scully MO, Zubairy MS (1997). Quantum Optics.

[44] Deng M (2020). On the interplay between physical and content priors in deep learning for computational imaging. Opt. Express.

[45] Zhang PL (2009). Improving resolution by the second-order correlation of light fields. Opt. Lett..

[46] Wang W (2015). Gerchberg-Saxton-like ghost imaging. Opt. Express.

[47] Mangeat T (2021). Super-resolved live-cell imaging using random illumination microscopy. Cell Rep. Methods.

[48] Yariv, A. & Yeh, P. *Photonics: Optical Electronics in Modern Communications* (Oxford: Oxford University Press, 2006).

[49] Healey GE, Kondepudy R (1994). Radiometric CCD camera calibration and noise estimation. IEEE Trans. Pattern Anal. Mach. Intell..

[50] Goodman, J. W. *Statistical Optics* (New York: Wiley-Blackwell, 2000).

[51] Howard, A. G. et al. MobileNets: efficient convolutional neural networks for mobile vision applications. Preprint at arXiv: 1704.04861v1 (2017).

[52] Glorot X, Bengio Y (2010). Understanding the difficulty of training deep feedforward neural networks. J. Mach. Learn. Res..

[53] Ruder, S. An overview of gradient descent optimization algorithms. Preprint at arXiv: 1609.04747v2 (2017).

[54] Ronneberger, O., Fischer, P. & Brox, T. U-Net: convolutional networks for biomedical image segmentation. *Proceedings of the 18th International Conference on Medical Image Computing and Computer-Assisted Intervention* (Munich, Germany: Springer, 2015).

