# Supplementary material for: Far-field super-resolution ghost imaging with a deep neural network constraint
Source: Light Sci Appl. 2022 Jan 1;11:1. doi: 10.1038/s41377-021-00680-w (PMC8720314; doi:10.1038/s41377-021-00680-w)
Supplement: Supplementary file 1 — Supplementary Material [file 41377_2021_680_MOESM1_ESM.docx]

**Supplementary Information for**

**Far-field super-resolution ghost imaging with a deep neural network constraint**

Fei Wang, Chenglong Wang, Mingliang Chen, Wenlin Gong

*Shanghai Institute of Optics and Fine Mechanics, Chinese Academy of Sciences, Shanghai, 201800, China*

*Center of Materials Science and Optoelectronics Engineering, University of Chinese Academy of Sciences, Beijing 100049, China*

Email: WangFei_m@outlook.com, wangchenglong@siom.ac.cn, cml2008@siom.ac.cn, gongwl@siom.ac.cn

Yu Zhang

*Shanghai Institute of Optics and Fine Mechanics, Chinese Academy of Sciences, Shanghai, 201800, China*

Email: yxu828@sina.com

Shensheng Han

*Shanghai Institute of Optics and Fine Mechanics, Chinese Academy of Sciences, Shanghai, 201800, China*

*Center of Materials Science and Optoelectronics Engineering, University of Chinese Academy of Sciences, Beijing 100049, China*

*Hangzhou Institute for Advanced Study, University of Chinese Academy of Sciences, Hangzhou 310024, China*

*CAS Center for Excellence in Ultra-intense Laser Science, Shanghai 201800, China*

Email: sshan@mail.shcnc.ac.cn

Guohai Situ*

*Shanghai Institute of Optics and Fine Mechanics, Chinese Academy of Sciences, Shanghai, 201800, China*

*Center of Materials Science and Optoelectronics Engineering, University of Chinese Academy of Sciences, Beijing 100049, China*

*Hangzhou Institute for Advanced Study, University of Chinese Academy of Sciences, Hangzhou 310024, China*

*CAS Center for Excellence in Ultra-intense Laser Science, Shanghai 201800, China*

Email: [ghsitu@siom.ac.cn](mailto:ghsitu@siom.ac.cn)

Tel: 021-69913671

Fax: 021-69918000


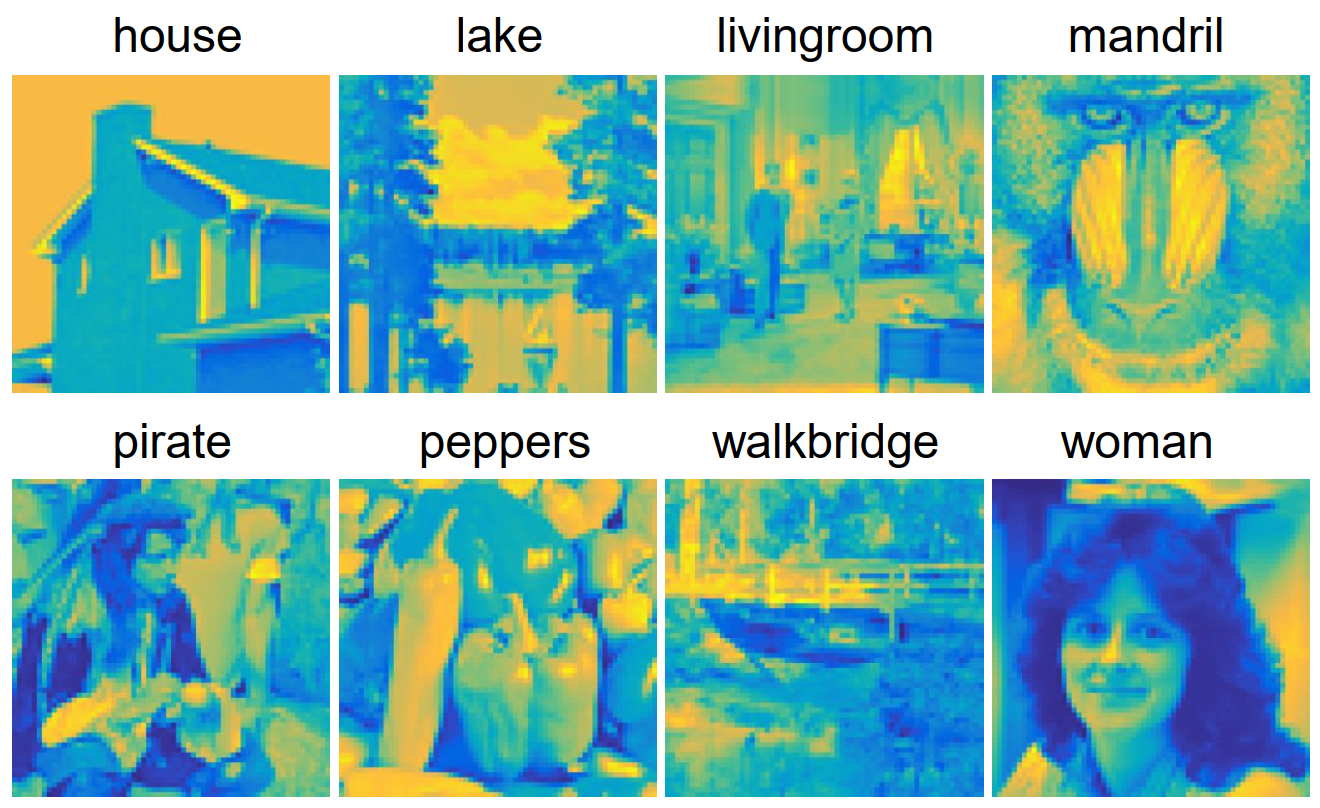


**Fig. S1** Standard grayscale test images used for analysis the robustness of GIDC. All the images were resized to 64$\times$64. The colormap is parula.


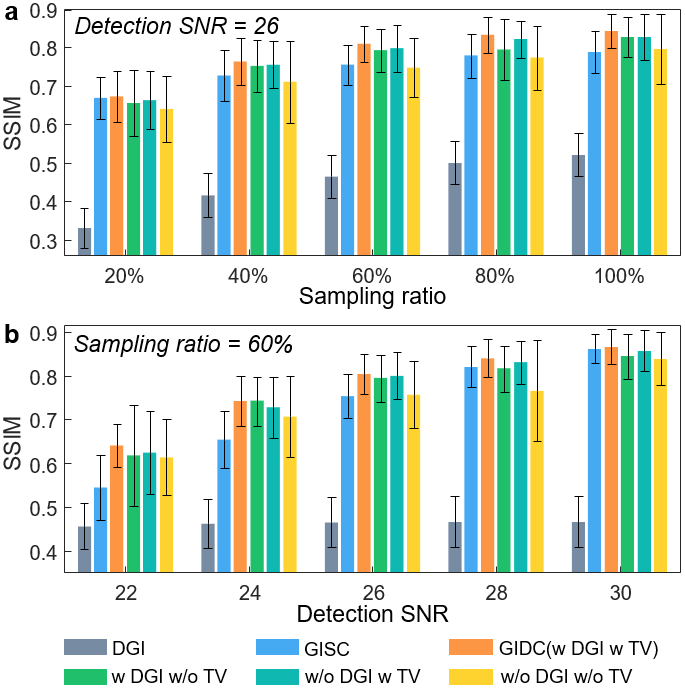


**Fig. S2** Quantitative analysis the effect of $\beta$ and dSNR on different reconstruction methods. For the GIDC-classes methods, when dSNR = 26 dB and $\beta$ is 20%, 40%, 60%, 80% and 100%, the iteration step is 1300, 1600, 1900, 2200 and 2500, respectively. When $\beta=60\%$ and the dSNR is 22 dB, 24 dB, 26 dB, 28 dB and 30 dB, the iteration step is 500, 1000, 1500, 2000 and 2500, respectively.


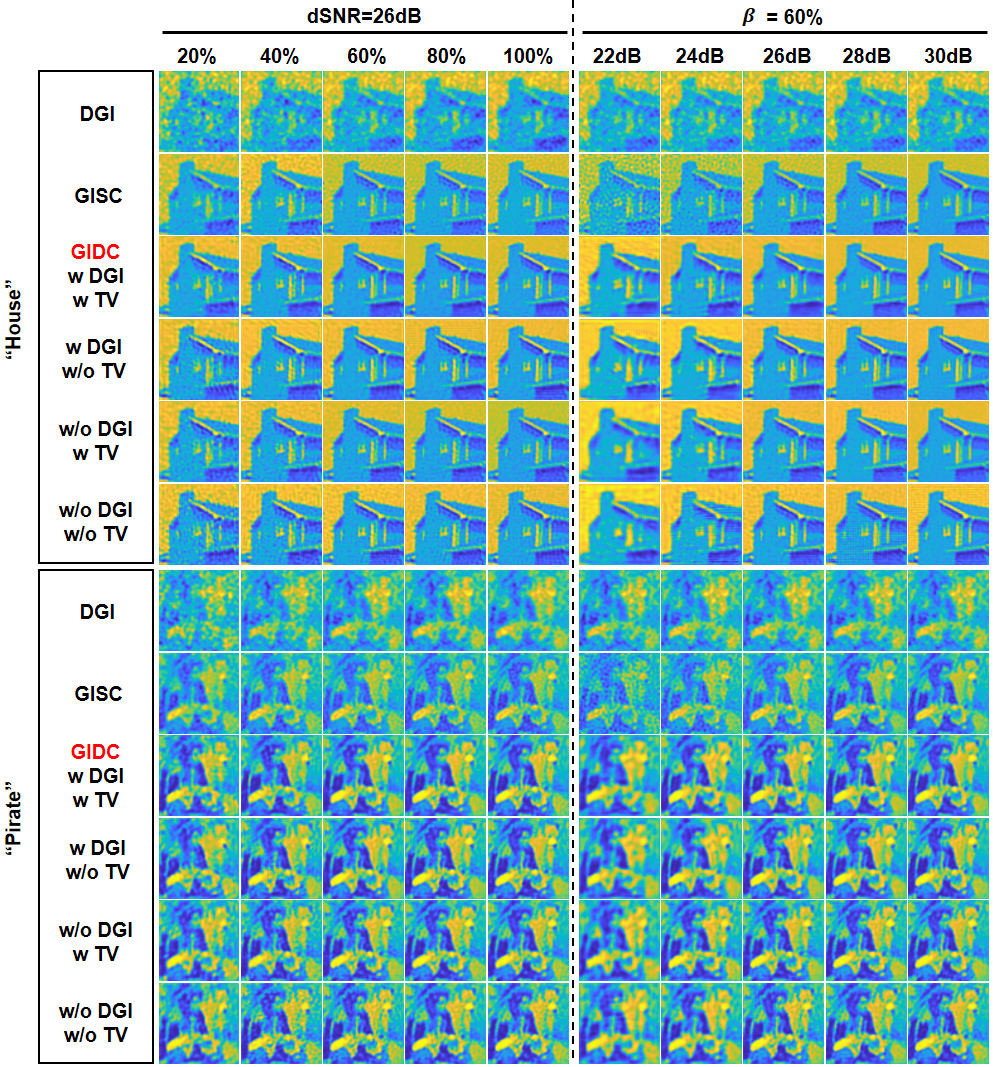


**Fig. S3** Visualization results of different GI reconstruction methods under different dSNR and $\beta$. For the sake of space, we only show the results on “House” and “Pirate”. We note that the other results follow similar trend.


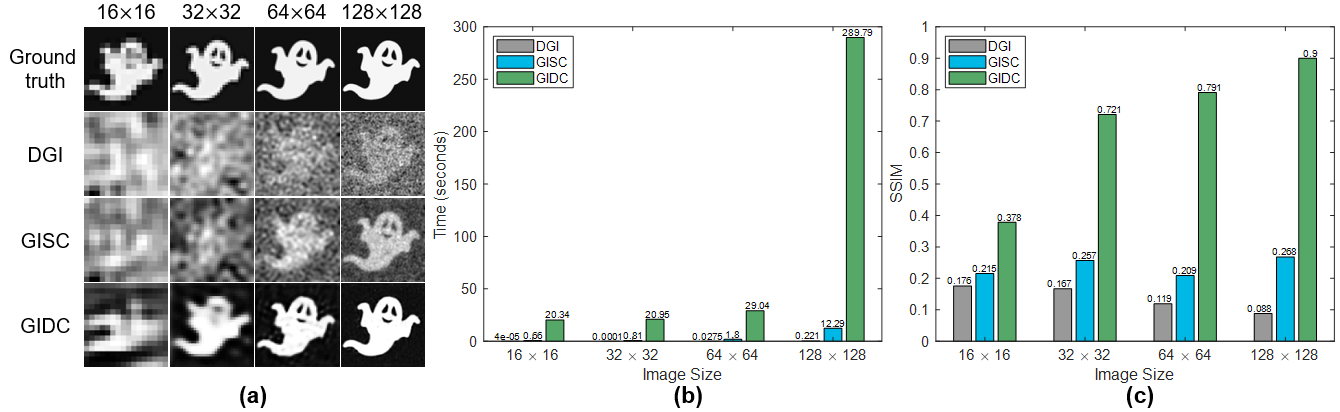
**Fig. S4** Comparison of computing time and reconstruction performance of DGI, GISC and GIDC when $\beta=6.25\%$ at different pixel resolutions. Visualization results of different reconstruction algorithms (a) and the corresponding computing time (b) and quantitative metrics (c).


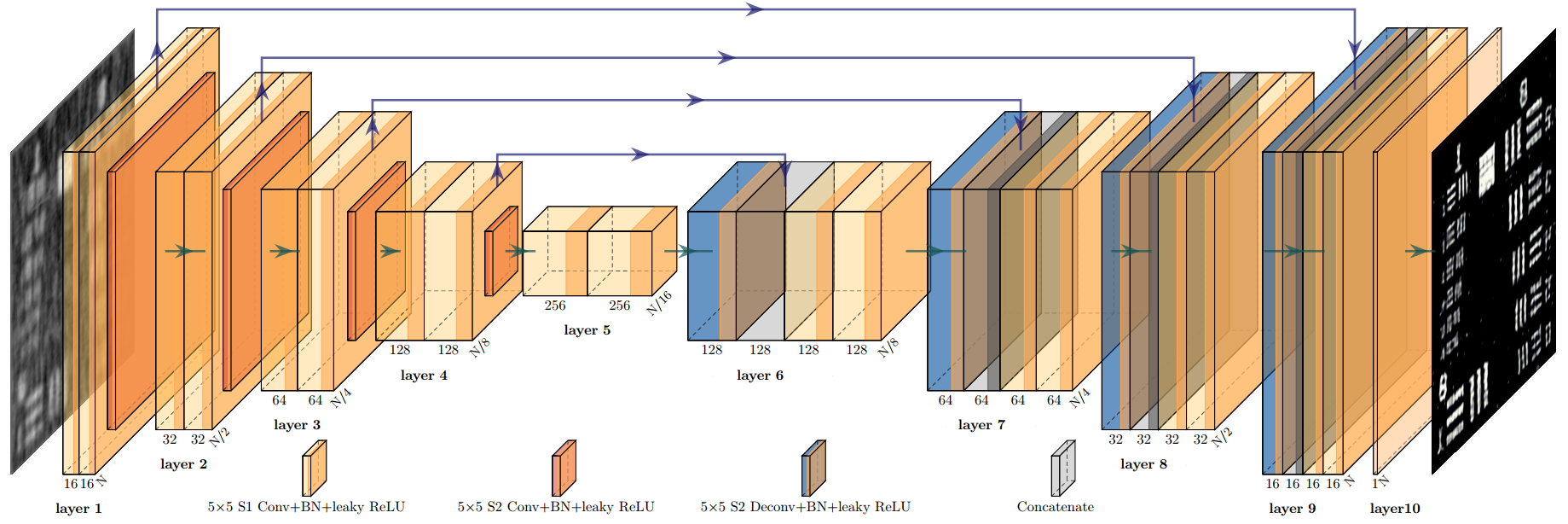


**Fig. S5** Diagram of neural network structure. Basically, it consists of an encoder path that takes the low quality result obtained by DGI as its input, a decoder path that outputs an estimated high quality GIDC result, and connections in the middle. It’s a pure convolution neural network, which uses convolution blocks 1 (5$\times$5 convolution (stride 1) + batch normalization + leaky ReLU), convolution blocks 2 (5$\times$5 convolution (stride 2) + batch normalization + leaky ReLU) and up-convolution blocks (5$\times$5 de-convolution (stride 2) + batch normalization + leaky ReLU). For the activation function in the output layer we use Sigmoid. We use the same neural network architecture for all the GI reconstruction tasks.
